# Properties and Structure of In Situ Transformed PAN-Based Carbon Fibers

**DOI:** 10.3390/ma11061017

**Published:** 2018-06-15

**Authors:** Jingjing Cao, Wenwu Zhao, Shuzhen Gao

**Affiliations:** College of Mechanical and Equipment Engineering, Hebei University of Engineering, Handan 056038, China; zhaowenwu1105@163.com (W.Z.); gaoshuzhen@163.com (S.G.)

**Keywords:** in situ transformed PAN-based carbon fibers, turbostratic structure, carbon layer, carbon atomic arrangement

## Abstract

Carbon fibers in situ prepared during the hot-pressed sintering in a vacuum is termed in situ transformed polyacrylonitrile-based (PAN-based) carbon fibers, and the fibrous precursors are the pre-oxidized PAN fibers. The properties and structure of in situ transformed PAN-based carbon fibers are investigated by Nano indenter, SEM, TEM, XRD, and Raman. The results showed that the microstructure of the fiber surface layer was compact, while the core was loose, with evenly-appearing microvoids. The elastic modulus and nanohardness of the fiber surface layer (303.87 GPa and 14.82 GPa) were much higher than that of the core (16.57 GPa and 1.54 GPa), and its interlayer spacing d002 and crystallinity were about 0.347 nm and 0.97 respectively. It was found that the preferred orientation of the surface carbon layers with ordered carbon atomic arrangement tended to be parallel to the fiber axis, whereas the fiber core in the amorphous region exhibited a random texture and the carbon atomic arrangement was in a disordered state. It indicates that the in situ transformed PAN-based carbon fibers possess significantly turbostratic structure and anisotropy.

## 1. Introduction

Carbon fiber has excellent properties, such as high strength, high modulus, low density, low thermal expansion, and high corrosion resistance [1], which makes it attractive for many applications. They include aircraft, medical equipment, construction, electronics, and machinery [2,3]. The important factors in selecting carbon fibers as reinforcement materials are their high strength and modulus, and to improve the mechanical properties of ceramic matrix composites, particularly their toughness [2]. Much research has been carried out to study the fabrication and mechanical properties of the short carbon fibers toughened ceramic matrix composites [4,5,6,7]. The commercial carbon fibers are usually chosen as the short carbon fibers, and its surface must be coated with epoxy resin slurry to protect it from damage. Therefore, chemical solvents need to be used to remove the slurry on the carbon fibers’ surface before use, and during removal, the chemical reaction may affect the surface quality of carbon fibers and degrades the carbon fibers’ properties [6], which results in a decrease in mechanical performance of the composites. Meanwhile, powder metallurgy process is used in the preparation of ceramic matrix composites, so carbon fibers will be easily damaged in the ball-milling process [7], which then affects its toughened efficiency in the composites. In addition, the commercial carbon fibers are more expensive. 

For commercial PAN-based carbon fibers, its fabrication process commonly involves stabilization and carbonization from PAN fibers. During the stabilization, the PAN molecules convert into ladder polymer by a series of cyclization, dehydrogenation, and oxidation reactions in air at below 270 °C under a certain tension. The tension is applied to prevent shrinkage of the PAN fibers. The ladder polymer is called pre-oxidized PAN fibers. After stabilization, the pre-oxidized PAN fibers are carbonized by heating in an inert atmosphere at 400~1500 °C, with no tension required. During carbonization, the ladder structure converts into a graphite-like structure by a cross-linking reaction. After carbonization, the graphitization is carried out by heating at 1500~3000 °C in an inert atmosphere, during which the crystallite size is increased and preferred orientation of carbon layer is improved. The inert gas (nitrogen or argon) is used to dilute the toxic waste gas generated in the process of carbonization and graphitization, which increases the production cost of PAN-based carbon fibers. Based on the fabrication process of PAN-based carbon fibers and the hot-pressed sintering process of ceramic, we propose to use pre-oxidized PAN fibers as precursors, which would be in situ transformed into PAN-based carbon fibers during the preparation of the composites in a vacuum. The hot-pressed sintering process not only provides the temperature required for carbonization and graphitization, but also contributes to the preferred orientation of carbon layers and regular arrangement of carbon atoms. The vacuum environment is beneficial to exhaust the toxic waste gas, as well as to prevent ingress of atmospheric air. Besides, the pre-oxidized PAN fibers have good flexibility to avoid damage in ball-milling and are cheap. This combination process has the advantages of simple process and low cost. Previous study had optimized the preparation process of in situ transformed PAN-based carbon fibers toughened alumina ceramic composites based on the thermal properties of pre-oxidized PAN fibers [8]. But the properties, preferred orientation of carbon layers, and regular arrangement of carbon atoms were not clear. Thus, the aim of this paper was to study the properties and structure of in situ transformed PAN-based carbon fibers, which will provide powerful data for further optimizing the preparation process of in situ transformed PAN-based carbon fibers toughened alumina ceramic composites and new ideas or methods for the preparation of other ceramic matrix composites.

## 2. Materials and Methods

### 2.1. Materials and Sample Preparation

The ceramic matrix powder selected for this study was α-Al_2_O_3_ powder (Zibo Rio Tinto Aluminum Co., Ltd., Zibo, China). The powder size was in a range of 2–10 μm, with purity and density of 99.8% and 3.96 g/cm^3^, respectively. Pre-oxidized PAN fibers (Bengbu Carbon Composite Co., Ltd., Bengbu, China) were used as the precursor of carbon fibers. The average diameter and density of the precursor fibers were about 14 μm and 1.4 g/cm^3^, respectively. 

The in situ transformed carbon fibers toughened alumina matrix composites were prepared in a vacuum using the hot-pressed sintering technology. The continuous pre-oxidized PAN fibers were chopped into 3~5 mm short fibers. Al_2_O_3_ powder and 25 vol. % chopped pre-oxidized PAN fibers were mixed and ball-milled, with 5 mm diameter alumina balls, for a lower attrition time to avoid damage to the chopped fibers. The mixtures were hot-pressed in a hexagonal boron nitride coated graphite die (placed in furnace (ZT-40-20Y, Chen Hua Technology Co., Ltd., Shanghai, China)) at 1700 °C under 40 MPa for 120 min in a vacuum of 1.3 × 10^−3^ Pa, and then cooled in the furnace to room temperature. The sintering process parameters for the composites were optimized [8,9], that is, grading heat preservation respectively at 444 °C and 1070 °C were maintained for 10 min to ensure that the cyclization and cracking reaction of pre-oxidized PAN fibers could be more completely and smoothly transformed into carbon fibers. And the heating rate was 10 °C/min. Initial pressure was maintained at 20 MPa before 1070 °C. At 1070~1400 °C, the pressure was slowly increased to 40 MPa with increasing temperature, and then the sintering pressure was kept invariant.

### 2.2. Characterization Methods

The cyclization degree of pre-oxidized PAN fibers was analyzed by Fourier Transformation Infrared (FT-IR) spectroscopy (Tensor 27, Bruker Corporation, Karlsruhe, Germany) with pressing potassium bromide troche. The pre-oxidized PAN fibers were cut and sifted using an 80 mesh sieve. The pellets were prepared by mixing 1 mg of chopped pre-oxidized PAN fibers with 100 mg of KBr. The mixture was then ground with an agate mortar. A hydraulic press was used to make the pellets by applying 10 MPa pressure and maintaining pressure for 10 min. Analyses were performed in the mid-infrared spectral region, from 4000 cm^−1^ to 400 cm^−1^ with a resolution of 2 cm^−1^. To characterize the microstructure and morphology of in situ transformed PAN-based carbon fibers, a variable-pressure field-emission scanning electron microscope (SEM; Supra 40VP, Zeiss Corporation, Oberkochen, Germany) was used. The specimens were coated with 10–20 nm of carbon to minimize charge, and the experimental conditions were set as 15 keV acceleration voltages and approximately 1 nA beam current. The elastic modulus and hardness of in situ transformed PAN-based carbon fibers were investigated by Nano mechanical properties microprobe (Nano Indenter XP, MTS Corporation, Minneapolis, MN, USA). The tests were performed at a 10 nm/s surface approach velocity, and the indentation depth was 500 nm. The Raman analysis was conducted with RM2000 Raman spectrometer (Renishaw, London, UK) using a laser wavelength of 514.5 nm (Argon ion) and fixed power at 4.7 mW. Lorentzian function was used for fitting the spectral lines. The crystallinity of in situ transformed PAN-based carbon fibers was evaluated by the integrated intensity ratio R of the D and G bands from the Raman spectra (R = I_D_/I_G_). An X-ray diffraction (XRD) of in situ transformed PAN-based carbon fibers was made with D/Max 2500 PC X-ray diffractometer (Rigaku Corporation, Tokyo, Japan). The measurements were carried out using Cu kα (λ = 0.154056 nm) and Ni filter at 0.02°/s scanning rate between 10°~75°. Using the Bragg formula, nλ = 2dsinθ, the interlayer spacing (d002) was determined, where θ is the Bragg angle (in rad). The analysis was performed using Origin software (Pro 8.0, OriginLab Corporation, Northampton, MA, USA) and Jade software (6.5, MDI Corporation, Livermore, CA, USA). And using Gaussian function, the peaks value of in situ transformed PAN-based carbon fibers was fitted. Both of the XRD and Raman testing samples were powdery obtained by crushing, grinding and sifting the composites. High resolution transmission electron microscopy (HR-TEM; JEM-2100, JEOL, Tokyo, Japan) was used to examine in situ transformed PAN-based carbon fibers, with acceleration voltage of 2000 kV. 

## 3. Results and Discussion

### 3.1. Cyclization Degree of Pre-Oxidized PAN Fibers

**Figure 1:**
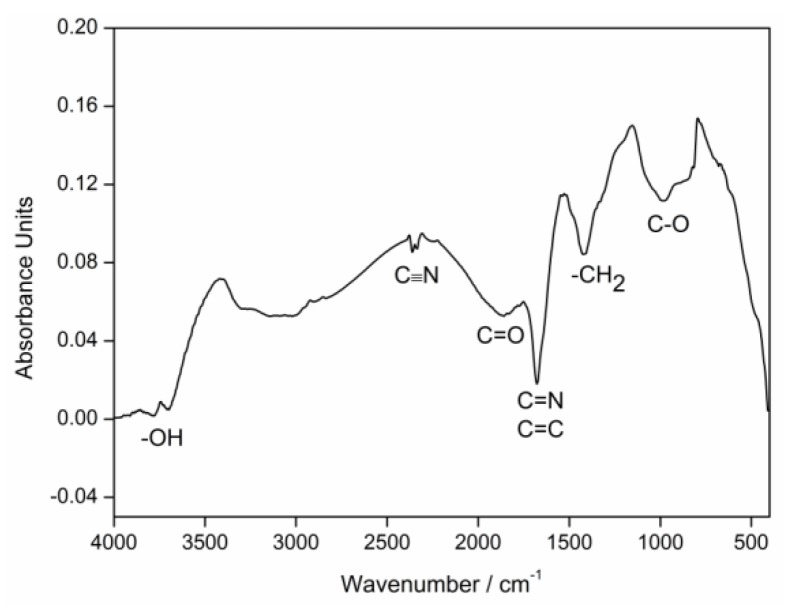
FT-IR spectrum of pre-oxidized PAN fibers.

The FT-IR spectrum of pre-oxidized PAN fibers is shown in Figure 1. The absorption band at 2248 cm^−1^ is attributed to the stretching and bonding vibrations of –C≡N cyano group, which is the characteristic absorption band of the PAN molecule [10]. It was due to the incomplete cyclization reaction that occurred in the PAN fibers during pre-oxidation stage and thus the residual PAN molecule is remained in the pre-oxidized PAN fibers. However, the absorption band intensity of –C≡N cyano group was weak, indicating that the pre-oxidation of PAN fibers was more complete. The weak absorption band at 3750 cm^−1^ and 1800 cm^−1^ indicated that –OH and C=O groups were formed in the oxidation reaction, also including the characteristic band of C–O group at 985 cm^−1^. The significant spectroscopic band at 1622 cm^−1^ was identified to be characteristic absorption bands of C=N and C=C group, whilst the peak localized at 1454 cm^−1^ was the bending and bonding vibration of –CH_2_ characteristic band. The C=N, C=C and –CH_2_ groups were characterized by characteristic absorption bands of heat-resistant trapezoidal structure formed during pre-oxidation of PAN fibers [11]. The FT-IR analysis indicated that the cyclization degree of PAN fibers was more complete, but a few PAN molecules still existed. Other bands may be attributed to the stretching and bonding vibration of absorbed and inner water. In addition, due to the low transparency of the pellet, light scattering can lead to baseline drift of the IR spectrum.

### 3.2. Microstructure of In Situ Transformed PAN-Based Carbon Fibers in the Composites

**Figure 2:**
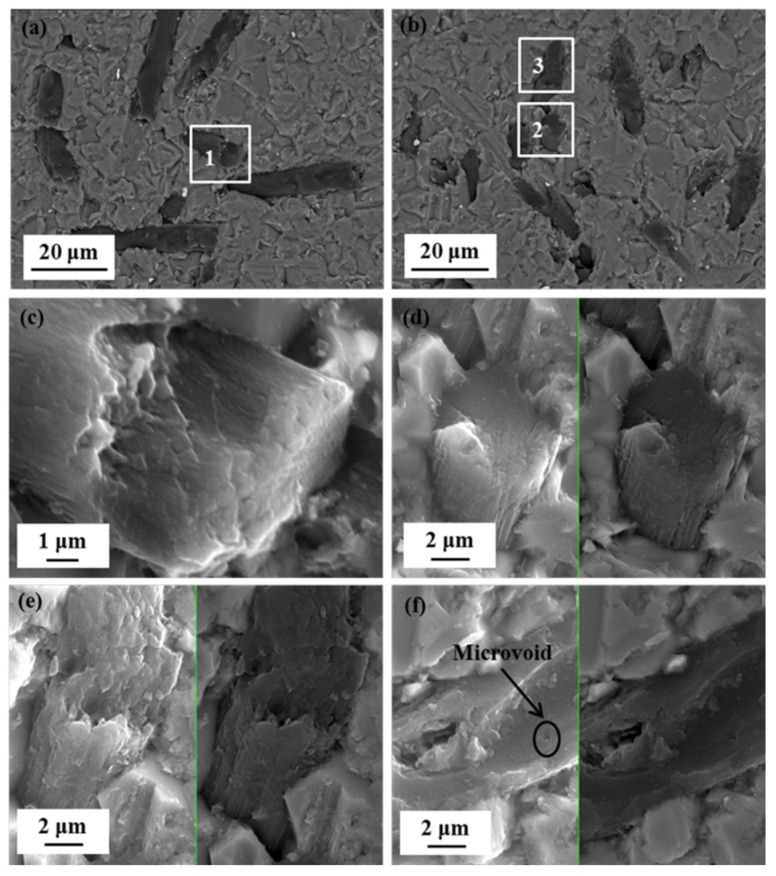
BSE (Backscattered Electron) and SE (Scattered Electron) images: (**a**) along the hot-pressed plane of the composites; (**b**) vertical to the hot-pressed plane of the composites; (**c**–**f**) in situ transformed PAN-based carbon fibers in the composites and (**c**–**e**) correspond to high magnification of the areas 1, 2, and 3 in (**a**,**b**).

The microstructures of the composites and in situ transformed PAN-based carbon fibers in the composites are displayed in Figure 2. Figure 2a,b shows the microstructure of the composites along the hot-pressed plane (perpendicular to the hot-pressed direction) and vertical to the hot-pressed plane (parallel to the hot-pressed direction), respectively. It can be found that the distribution of in situ transformed PAN-based carbon fibers in the composites was basically perpendicular to the hot-pressed direction, suggesting that the in situ transformed PAN-based carbon fibers in the composites were preferred orientation distribution along the hot-pressed plane. Figure 2c–f show the microstructure of in situ transformed PAN-based carbon fibers in the composites, and Figure 2c–e images represent, respectively, the white square areas marked as 1, 2, and 3 in Figure 2a,b. From Figure 2c,d, it is observed that the microstructure of in situ transformed PAN-based carbon fibers from surface to core were compact, with an obvious lamellar structure in which the carbon layers were parallel to the fiber axis (Figure 2c). Some in situ transformed PAN-based carbon fibers showed loose microstructure (Figure 2e), with even microvoids occurring in the core as shown in Figure 2f. Those microvoids were generated mainly due to the elimination of the gases. From the FT-IR spectrum (Figure 1), there was a large number of non-C atoms such as N, H, and O in the pre-oxidized PAN carbon fibers, that escaped in the form of gases (N_2_, H_2_, NH_3_, H_2_O, et al.) owing to the polycondensation reaction during the carbonization (or during the hot-pressed sintering). Microvoids will be formed in the in situ transformed PAN-based carbon fibers if these gases cannot escape from the fibers [12]. High pressure and vacuum were beneficial to eliminate the gases from the fibers, and hence reduce the amount of microvoids [13]. It is reported that microvoids can affect the properties of PAN-based carbon fibers, which will be studied next.

### 3.3. Elastic Modulus and Hardness of In Situ Transformed PAN-Based Carbon Fibers

**Figure 3:**
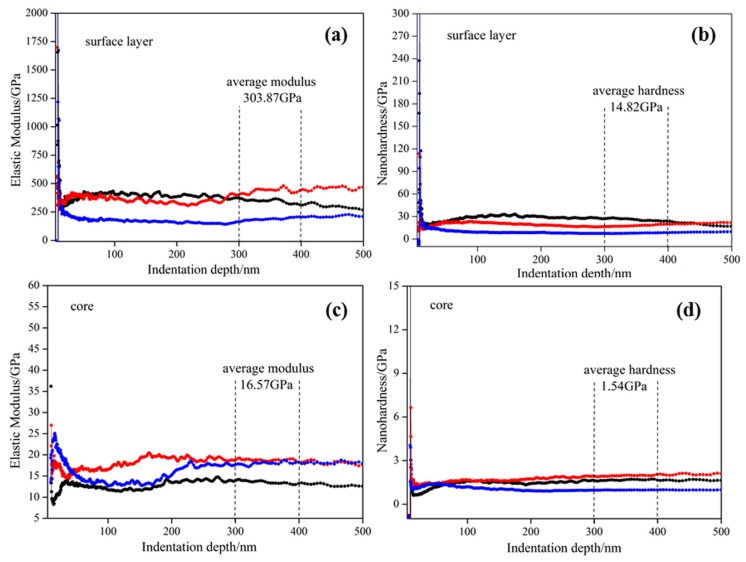
Elastic modulus-depth and nanohardness-depth curves of in situ transformed PAN-based carbon fibers: (**a**) elastic modulus-depth curve of the fiber surface layer; (**b**) nanohardness-depth curve of the fiber surface layer; (**c**) elastic modulus-depth curve of the fiber core; (**d**) nanohardness-depth curve of the fiber core.

Properties of in situ transformed PAN-based carbon fibers in the composites were tested using nano indentation. The surface layers properties were tested on the planes (Figure 2a), which were perpendicular to the hot-pressed direction, and its core properties were tested on the planes (Figure 2b), which were parallel to the hot-pressed direction. Three points were selected arbitrarily from both the surface layers and the core to measure their elastic modulus and nanohardness. The elastic modulus-indentation depth and the nanohardness-indentation depth curves of in situ transformed PAN-based carbon fibers are shown in Figure 3. It can be seen that the deviations of the curves are small with increasing indentation depth, suggesting a homogeneous property of in situ transformed PAN-based carbon fibers. To characterize the average values of elastic modulus and nanohardness, the data were calculated from the indentation depth between 300 nm and 400 nm. The elastic modulus and nanohardness of the fiber surface layer were estimated to be 303.87 GPa and 14.82 GPa respectively, as in Figure 3a,b, while the two value of the fiber core were calculated as 16.57 GPa and 1.54 GPa respectively shown in Figure 3c,d. The results indicate that the in situ transformed PAN-based carbon fibers have significant anisotropic [3,14], exhibiting better properties in the fiber surface layers than the fiber core. The poor properties in the fiber core were likely associated with its loose microstructure and the presence of microvoids seen in Figure 2e,f. Therefore, the significant differences in properties of in situ transformed PAN-based carbon fibers were mainly related to its structure and the arrangement of carbon atoms.

### 3.4. Structure of In Situ Transformed PAN-Based Carbon Fibers

**Figure 4:**
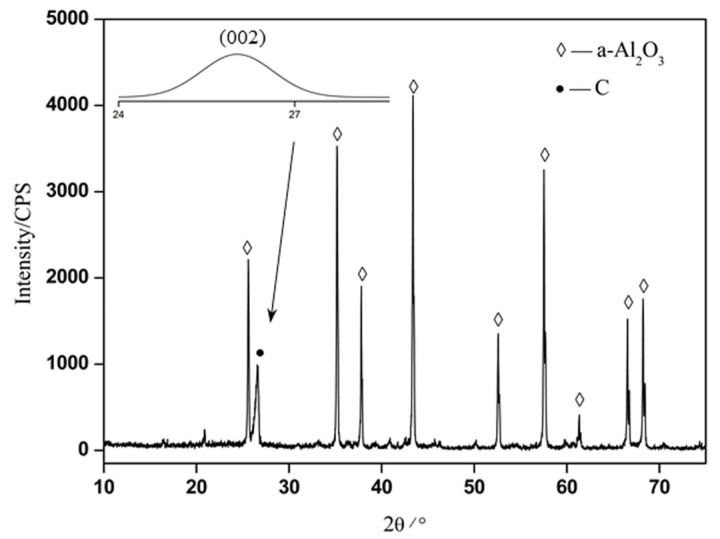
XRD pattern of the composites.

XRD was utilized to analyze the phase structure of in situ transformed PAN-based carbon fibers in composites. The XRD pattern of the composites was shown in Figure 4, which shows obvious diffraction peaks of α-Al_2_O_3_ phase. The diffraction peak at about 26° was C phase, corresponding to the (002) crystal face of in situ transformed PAN-based carbon fibers. Using a Gaussian function, the in situ transformed PAN-based carbon fibers was fitted and the fitting chart is shown in Figure 4. According to the Bragg formula, the interlayer spacing (d002) of in situ transformed PAN-based carbon fibers was calculated as approximately 0.3422 nm.

**Figure 5:**
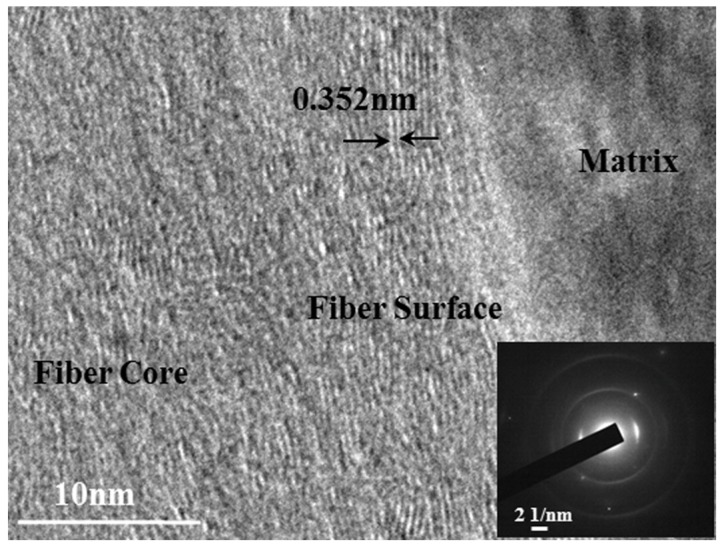
HR-TEM image and diffraction pattern of in situ transformed PAN carbon fibers.

The HR-TEM image and electron diffraction pattern of in situ transformed PAN-based carbon fibers in composites is shown in Figure 5. It is noticed that the dark gray region on the right is a matrix and the white gray region on the left is in situ transformed PAN-based carbon fibers. There appears to be a large number of white stripes, which were carbon layers of in situ transformed PAN-based carbon fibers. The carbon layers in the fiber surface tend to have the preferred orientation parallel to the fiber axis, whereas the carbon layers in the fiber core exhibited a random texture. Moreover, the carbon layers of the surface were not completely flat, but slightly bent, forming a certainly preferred orientation along the fiber axis, while the carbon layers of the core were unclear and crooked or crossed/merged together. As a result, the in situ transformed PAN-based carbon fibers had a significant turbostratic structure.

The interlayer spacing (d002) of in situ transformed PAN-based carbon fibers was approximately 0.352 nm measured using the digital micrograph software. With consideration of the calculated result of Bragg formula, the average value of interlayer spacing d002 was approximately 0.347 nm, which was almost identical to the typical HS-typed PAN based carbon fibers, with an interlayer spacing d002 value of 0.348 nm [15]. Harald [16] conducted statistics for different PAN-based carbon fibers and obtained a mean value of d002 as 0.353 nm. All the above values were larger than the interlayer spacing (d002 = 0.3354 nm) of the ideal graphite crystal, due to the turbostratic structure of in situ transformed PAN-based carbon fibers. In addition, the preparation temperature (1700 °C) of in situ transformed PAN-based carbon fibers was lower than the graphitization temperature (2000~2400 °C) of PAN-based carbon fibers. At lower temperature, orientation of the carbon layers and arrangement of carbon atoms become less ordered, which caused larger interlayer spacing.

In Figure 5, a bright diffraction arc and two weak diffraction rings were displayed. The bright diffraction arc implies that in situ transformed PAN-based carbon fibers contained crystalline structure corresponding to the (002) crystal plane of carbon fibers. And the two diffraction rings suggest that the in situ transformed PAN-based carbon fibers also contained amorphous structure corresponding to the (100) and (110) crystal planes respectively. It again indicates that the structure of in situ transformed PAN-based carbon fibers consist of both crystalline regions and amorphous regions, exhibiting the turbostratic nature. 

**Figure 6:**
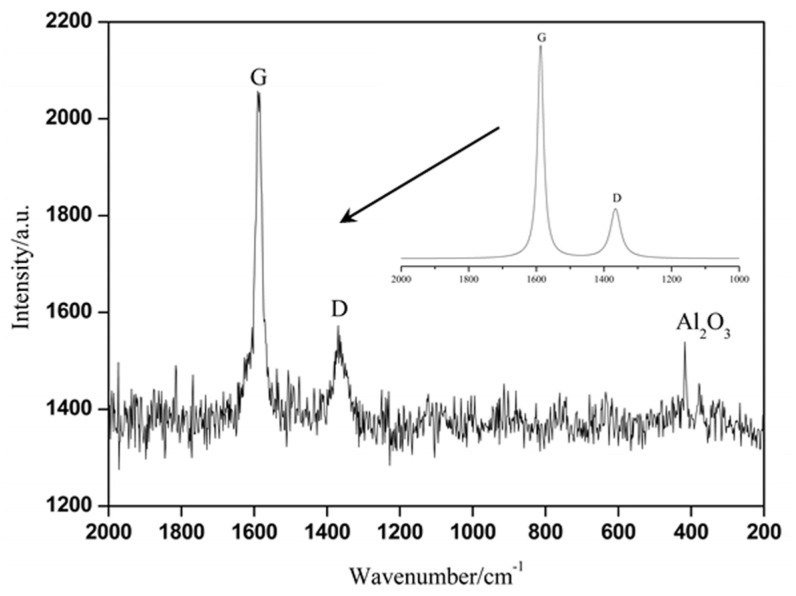
Raman spectrum of the composites.

Raman scattering is an effective way of quantitative analysis for amorphous or disordered existing in the structure of carbon materials [2]. Raman spectrum of the composites is displayed in Figure 6, which showed two spectral peaks at about 1587.5 cm^−1^ and 1365.5 cm^−1^, respectively. It was reported that carbon materials with graphite-like structure generally have two strong characteristics spectral peaks at 1580 cm^−1^ and 1360 cm^−1^, namely G peak and D peak [17]. It reveals that the spectral peaks of 1587.5 cm^−1^ and 1365.5 cm^−1^ in Figure 6 can be referred to respectively as G peak and D peak. The Raman spectral peak at about 400 cm^−1^ was the characteristic spectral peak of alumina.

The Raman spectrum of the studied in situ transformed PAN-based carbon fibers between 1000 cm^−1^ and 2000 cm^−1^ was fitted using Lorentzian function and the rebuilt fitting spectrum was also illustrated in Figure 6, showing two peaks at 1587.5 cm^−1^ (G peak) and 1365.5 cm^−1^ (D peak). The G peak was related to vibration of the C–C bond with sp^2^ hybridized carbon atoms in the six-membered ring, and its intensity represents the ordered C–C bond structure in the graphite [2,18]. And the D peak was generated by telescopic shock of bonds with sp^3^ hybridized at the grain boundary or other defects, and its intensity represents the disorder existing in the graphite structure [2]. To evaluate the crystallinity of in situ transformed PAN-based carbon fibers, the intensity ratio R (I_D_/I_G_) of the G and D peaks is used. The R value of in situ transformed PAN-based carbon fibers was 0.97 under experimental conditions, which was more than twice the typical value of commercial PAN-based carbon fibers (0.42). The increase of R value indicates the decrease of crystallinity [1,2,18,19], which means that the order degree of in situ transformed PAN-based carbon fibers was lower, due to a large fraction of turbostratic carbon phase (disordered carbon phase) in it. 

The combined analysis results from the XRD and TEM confirmed that the carbon layers on the fiber surface had preferred orientation along the fiber axis and parallel to one another, but not completely parallel to the fiber axis or stacked in any particular sequence [1]. Yet, the carbon layers in the fiber core were not even parallel to one another. And the carbon atomic arrangements of the surface layers in the crystalline regions are in order, whereas the carbon atomic arrangements of the core in the amorphous regions were in disorder. Besides, the reorganization of carbon atomic during carbonization (or during hot-pressed sintering) causes the fibers to shrink and when the arrangement of carbon atoms is disturbed, microvoids were generated resulting in loose tissue and lower performance. Nevertheless, with regular arrangement of carbon atoms, microstructure of the fiber in the surface layers was denser with significantly stronger mechanical properties in comparison with those of the core, which showed looser microstructure with possible microvoids (Figure 2), and lower elastic modulus and hardness properties (Figure 3). As a main factor affecting the fiber properties, the differences in the structure of in situ transformed PAN-based carbon fibers made it have significant anisotropy.

## 4. Conclusions

From the above analysis results, the main conclusions can be drawn as follows:

(1) The in situ transformed PAN-based carbon fibers had an obvious turbostratic structure, and the microstructure of the fiber surface layer was compact, while the microstructure of the core was loose and even appeared to have microvoids.
(2) The elastic modulus and nanohardness of the in situ transformed PAN-based carbon fiber surface layers (303.87 GPa and 14.82 GPa, respectively) were much higher than that of the core (16.57 GPa and 1.54 GPa, respectively).
(3) The XRD pattern showed a characteristic diffraction peak of the carbon fiber at about 26°, which corresponded to (002) crystal face of the carbon fibers and its interlayer spacing d002 was approximately 0.347 nm. In Raman spectrum, there were characteristic spectral peaks (G peak and D peak) of in situ transformed PAN-based carbon fibers and its degree of crystallinity was 0.97. And the crystalline regions correspond to the (002) crystal plane of in situ transformed PAN-based carbon fibers surface layers, whereas the amorphous regions correspond to (100) or (110) crystal plane of the fiber core. The surface layers tended to have the preferred orientation of carbon layers parallel to the fiber axis and ordered arrangement of carbon atoms, whereas the core tended to exhibit a random texture and disordered carbon atoms.

It is concluded that the loose microsturcture, random texture, order degree of carbon atomic arrangement, interlayer spacing, and crystallinity of the carbon fibers all affected its properties. The elastic modulus of in situ transformed PAN-based carbon fibers was relatively lower than that of the commercial HM typed PAN-based carbon fiber (350–700 GPa). Therefore, it is the key to improve the orientation of carbon layers parallel to the fiber axis and the carbon atomic arrangement of carbon fiber core in order to obtain high performance carbon fibers, which is the attentive focus of our next work.
